# Deferiprone Rescues Behavioral Deficits Induced by Mild Iron Exposure in a Mouse Model of Alpha-Synuclein Aggregation

**DOI:** 10.1007/s12017-017-8447-9

**Published:** 2017-06-16

**Authors:** Eleonora Carboni, Lars Tatenhorst, Lars Tönges, Elisabeth Barski, Vivian Dambeck, Mathias Bähr, Paul Lingor

**Affiliations:** 1 Department of Neurology, University Medicine Göttingen, Robert-Koch-Str. 40, 37075 Göttingen, Germany; 2 Cluster of Excellence Nanoscale Microscopy and Molecular Physiology of the Brain (CNMPB), Göttingen, Germany; 3 Department of Neurology, Ruhr-University Bochum, Bochum, Germany

**Keywords:** Alpha-synuclein, Iron, Deferiprone, Parkinson’s disease, Rotarod, Novel object recognition test

## Abstract

Parkinson’s disease (PD) is the most common neurodegenerative movement disorder, and its causes remain unknown. A major hallmark of the disease is the increasing presence of aggregated alpha-synuclein (aSyn). Furthermore, there is a solid consensus on iron (Fe) accumulation in several regions of PD brains during disease progression. In our study, we focused on the interaction of Fe and aggregating aSyn in vivo in a transgenic mouse model overexpressing human aSyn bearing the A53T mutation (prnp.aSyn.A53T). We utilized a neonatal iron-feeding model to exacerbate the motor phenotype of the transgenic mouse model. Beginning from day 100, mice were treated with deferiprone (DFP), a ferric chelator that is able to cross the blood–brain barrier and is currently used in clinics as treatment for hemosiderosis. Our paradigm resulted in an impairment of the learning abilities in the rotarod task and the novel object recognition test. DFP treatment significantly improved the performance in both tasks. Although this was not accompanied by alterations in aSyn aggregation, our results support DFP as possible therapeutic option in PD.

**Electronic supplementary material:**

The online version of this article (doi:10.1007/s12017-017-8447-9) contains supplementary material, which is available to authorized users.

## Introduction

Parkinson’s disease (PD) is the most common neurodegenerative movement disorder (Pringsheim et al. 2014). PD pathogenesis is still poorly understood, but it is thought to be a multifactorial disease with no curative therapy available (Kalia and Lang 2015). Aging is regarded as a major risk factor and, in a society with increasing life expectancy, PD represents a massive socioeconomic burden, where new treatment approaches are urgently needed.

Despite being mostly idiopathic, rare hereditary forms of PD exist (Singleton et al. 2013). Duplications, triplications as well as single point mutations in the genetic sequence of alpha-synuclein (aSyn) have been found in families with inherited forms of PD (Kasten and Klein 2013; Polymeropoulos et al. 1997). Lewy bodies (LB) represent a hallmark of the disease in both idiopathic and some hereditary forms, and they consist mainly of aSyn aggregates (Spillantini et al. 1997), which underlines a prominent role of this protein in PD etiology.

Another key factor in PD is the accumulation of iron (Fe), which occurs mostly in the substantia nigra pars compacta (SNpc) (Dexter et al. 1989). This is of interest, since PD is characterized by particular vulnerability of dopaminergic neurons in this specific brain region that is responsible for many motor symptoms of the disease. The central role of Fe is furthermore underlined by its co-localization in LB (Castellani et al. 2000). Beside histological studies, also NMR analyses could demonstrate a direct interaction between aSyn and Fe, increasing the protein’s aggregation propensity (Binolfi et al. 2006; Golts 2002; Peng et al. 2010). Thus, Fe dyshomeostasis represents an important feature in the pathogenesis of PD that needs a deeper understanding in order to enter clinical trials using Fe chelators in addition to the symptomatic treatment (Devos et al. 2014).

Among chelators, deferiprone (DFP) is particularly interesting. In fact, it is a bidentate ferric chelator that is able to cross the blood–brain barrier (Fredenburg et al. 1996). It has been successfully used clinically for more than two decades for Fe-storage pathologies such as neurodegeneration with brain iron accumulation (Abbruzzese et al. 2011) and Friedreich’s ataxia (Elincx-Benizri et al. 2016), and it is currently employed as treatment for Fe overload in thalassemia major (Perez et al. 2008). DFP is an intriguing drug in its category as it chelates Fe in a more conservative fashion by repositioning it to the sites of use (Cabantchik et al. 2013). This means that DFP can chelate the excess of Fe in the intracellular labile Fe pool and then redistribute it to extracellular apotransferrin (Sohn et al. 2011), by that reducing an excessive Fe loss in the organism. In previous studies, DFP treatment increased the survival of dopaminergic neurons in the SNpc in toxin-induced animal models of PD using 6-hydroxydopamine (6-OHDA) or 1-methyl-4-phenyl-1,2,3,6-tetrahydropyridine (MPTP) (Devos et al. 2014; Workman et al. 2015).

The aim of the present study was to analyze the interplay of Fe and aSyn in vivo. To that, a transgenic mouse model of aSyn aggregation (Giasson et al. 2002) underwent Fe supplementation and the role of Fe chelation was evaluated by DFP administration.

## Methods

### Fe Intoxication and DFP Treatment

The transgenic mouse line B6;C3-Tg(Prnp-SNCA*A53T)83Vle/J (short: A53T mice) was first described by Giasson et al. (2002). Heterozygous breeding couples were purchased from Jackson Labs (J004479; Bar Harbor, ME) and mice were bred in the Central Animal Care Unit of the University Medicine Göttingen, Germany. The animals were treated according to the regulations of the local animal research council and legislation of the State of Lower Saxony, Germany (33.12-42502-08-13/1233). This mouse line expresses the human sequence of aSyn bearing the A53T mutation that is responsible for a familial form of PD (Polymeropoulos et al. 1997) under the prion protein promoter. The phenotype of this line is represented by the onset of motor symptoms (also addressed as “disease onset”) ranging from postnatal day (p) 250–550 (Giasson et al. 2002). Mice display cognitive decline and severe motor impairment, ultimately leading to paralysis and death. In the present study, mild Fe exposure was produced according to the protocol of Fredriksson and colleagues (Fredriksson et al. 1999) using 40 mg/kg body weight (bw) of Fe carbonyl (#C3518, Sigma-Aldrich, Darmstadt, Germany) dispersed in 5% sorbitol (#S1876, Sigma-Aldrich) applied daily to mouse pups via oral gavage between p10 and p17. Special tubes for mouse pups were used (#FTP22-25, Instech Laboratories, Plymouth Meeting, PA). Control mice received vehicle only (5% sorbitol). The oral chelator DFP (Apopharma Inc., Rockville, MD) was given via the drinking water at a concentration of 50 mg/L starting from p100 until sacrifice. Based on a daily drinking volume of 6 mL and a mean weight of 30 g per mouse, the daily dosage of DFP was considered to be of 10 mg/kg bw per mouse.

The transgenic animals were divided into 4 different treatment groups: “A53T/Veh” animals received only vehicle; “A53T/Fe” animals received only Fe carbonyl; “A53T/DFP/Veh” animals received vehicle and DFP supplement in the drinking water; “A53T/DFP/Fe” animals received Fe carbonyl and DFP supplement in the drinking water. For each group, 10 animals were sacrificed at the onset of the symptoms (“disease onset”) and five animals were sacrificed at p250. In addition to transgenic mice, also wild-type (WT) littermates were added in the study in order to investigate the effects of Fe supplementation in animals with WT genetic background: “WT/Veh” were mice that received vehicle, and “WT/Fe” were animals that received the same paradigm of Fe supplementation as the transgenic littermates.

Animals were weighed weekly to evaluate the general condition of health and also to determine the onset of the disease, which is accompanied by weight loss in A53T animals (Giasson et al. 2002). Mice were sacrificed when there was a weight loss of 20%, or when the rotarod performance of the animal dropped to 50% of the average of the previous performances.

### Rotarod Test

Rotarod was performed on transgenic animals as previously described (Jones and Roberts 1968; Tatenhorst et al. 2016) using a rotarod for mice (#47600, Ugo Basile, Comerio, Italy) where 5 mice could perform the test simultaneously. The animals were pre-trained on 3 consecutive days at 10 rpm for 5 min. Mice were tested once a week from p220 onward at an accelerating speed ranging from 5 to 40 rpm within 5 min. Each test consisted of three repetitions with an inter-trial interval of 30 min in order to reduce stress and fatigue, and the means from these three runs were analyzed. Each performance was recorded as the time in seconds spent on the rotating rod until the animal fell off or until the end of the task. The “disease onset” of the transgenic mice rotarod was determined when the animals performed worse than 50% of the long-term average. One-way ANOVA with repeated measures was used for statistical purposes.

### Novel Object Recognition (NOR) Test

The novel object recognition test assesses the presence of cognitive deficits in animals especially regarding memory and learning. The test was performed at p250 and at disease onset as previously described (Bevins and Besheer 2006; Tatenhorst et al. 2016). Briefly, a single mouse was placed for 3 min inside an arena of 48 × 35 cm to habituate. Then, two identical objects were placed inside the arena, and the mouse was left for 5 min to familiarize with the objects. The mouse was inserted back in its home cage for 10 min and subsequently placed again in the arena for 4 min with one familiar and one completely novel object. The test was digitally recorded and later evaluated by a blinded investigator using EthoVision XT 8.5 software (Noldus, Groningen, the Netherlands). The time spent with the familiar object and the novel object was recorded as well as the distance moved and the movement velocity. The discrimination ratio was calculated as described before (Bevins and Besheer 2006; Tatenhorst et al. 2016) and analyzed between the treatment groups. Statistical analysis was done comparing animals with the same genetic background.

### Catwalk™ Gait Analysis

To assess the gait performance, the Catwalk™ XT gait analysis system (Noldus) was utilized. The test was performed at p250 as well as at disease onset as described before (Saal et al. 2015; Tatenhorst et al. 2016). The animals were placed in a walkway of 4 cm width with a glass bottom and recorded by a high-speed digital camera from below. The footprints were automatically detected by the Catwalk™ XT 10.0 software. Detection settings were as follows: camera gain 20; intensity threshold 0.10; max. allowed speed variation 60%. Three compliant runs per animal were recorded, and the means out of these runs were analyzed over the treatment groups. In the current study, we focused on gait parameters which were described to be altered in the A53T mouse model before: print area, swing speed, stride length and step sequence regularity index (Tatenhorst et al. 2016). The data were analyzed by a MATLAB script-calculated one-way ANOVA for all the parameters on all the groups at the two different time points.

### Animal Sacrifice and Tissue Processing

At p250, or at disease onset, animals were sacrificed by exsanguination: under deep anesthesia, mice were transcardially perfused with PBS. The whole brain was removed from the cranial cavity, and the two hemispheres were separated. One hemisphere was fixed in 4% PFA overnight for subsequent histological evaluations. The other hemisphere was sectioned into four regions: cortex, cerebellum, hippocampus and midbrain. The tissue was flash frozen in liquid nitrogen and stored at −80 °C until further protein analyses.

### Proteinase K Digestion and Dot Blot

Proteinase K digestion was carried out in order to assess the amount of aSyn aggregates in the postmortem brains of the A53T animals. All chemicals were purchased from Sigma-Aldrich, Darmstadt, Germany, unless otherwise stated. The digestion was accomplished as described before (Butler et al. 1988). Briefly, the brain tissue was homogenized with four volumes of homogenization buffer (0.05 M Tris hydrochloride pH 7.5, 0.15 M NaCl, 5 mM EDTA) at 4 °C by using a bead mill homogenizer (Precellys 24, Peqlab, Erlangen, Germany); then, proteins were extracted by adding 1% Triton X-100 and 1% deoxycholate to the homogenates. A centrifugation at 5000 g for 10 min permitted the removal of nuclei and insoluble debris. The proteins in the supernatant were precipitated with 4 volumes of methanol for 30 min at −20 °C. The precipitate was collected by centrifugation at 5000 g for 30 min.

The brain extracts were re-suspended in one volume of re-suspension buffer (0.05 M Tris hydrochloride pH 7.5, 0.15 M NaCl, 0.2% sarkosyl), and the proteins were quantified via BCA assay (Thermo Fisher, Waltham, MA). To 22 mg of total protein extracts, 3.3 µAU of proteinase K (Applichem, Darmstadt, Germany) was added, adjusting the volume to 50 µL. Digestions were carried out at 37 °C for 10 min, and the reaction was stopped by adding 5 mM PMSF to inhibit proteinase K enzymatic activity. The samples were added with 5x sample buffer, boiled at 95 °C for 5 min and subsequently used for dot blot analysis.

Dot blots were performed as previously described (Oprandy et al. 1988) in order to visualize all possible proteinase K-resistant forms of aSyn, by using a Minifold I system (Sigma, #WHA10447850). Ten micrograms of each sample were  added to the sample buffer for the dot blot up to 400 µL final volume and run through the wells by applying vacuum. Then, the membrane was blocked for one hour in 5% dry skimmed milk (Applichem) in TBS-T. The primary antihuman aSyn antibody (dilution 1:500, #32-8100, Invitrogen, Carlsbad, CA) was then incubated in 2.5% skimmed milk in TBS-T at 4 °C overnight. The membrane was washed 5 times for 10 min with TBS-T; then, the secondary antibody conjugated horseradish peroxidase anti-mouse (Dako, Jena, Germany, dil. 1:2000) was applied for 1 h at room temperature. Chemiluminescence signal was visualized using ECL (Immobilon Western, Millipore, Billerica, MA) on X-ray films (GE Healthcare, Amersham, UK) and analyzed using ImageJ 1.47v (National Institutes of Health, Bethesda, MD).

### Immunohistochemical Staining

For immunohistochemical analysis, all chemicals were purchased from Sigma-Aldrich, unless otherwise stated. Brain hemispheres were fixed in 4% PFA/PBS overnight, afterward cryoprotected in 30% sucrose (Applichem) in PBS for 24 h at 4 °C, snap-frozen and stored at −80 °C until sectioning. Coronal cryosections (30 µm) were collected and kept in 0.1% sodium azide/PBS. On demand, sections were mounted on slides, dried for 1 h at 37 °C and then overnight at RT. For immunohistochemical staining, sections were rehydrated for 1 h in PBS and then incubated for 30 min in citrate buffer (10 mM citric acid, 0.05% Tween 20, pH 6.0) at 80 °C. After cooling down, sections were washed with PBS, followed by incubation in 0.1 g Sudan black (Applichem) per 100 ml 70% EtOH. After rinsing with water and PBS washing, sections were incubated for 20 min in 25 mM glycine (Applichem) in PBS. Afterward, sections were blocked for 1.5 h in a solution of 10% normal horse serum (NHS, PAA), 5% bovine serum albumin (BSA, Applichem), 0.3% Triton X-100 (Applichem) and 25 mM glycine in PBS. First antibodies against p-S129-aSyn (# ab59264, Abcam, Cambridge, UK, dil. 1:100) and tyrosine hydroxylase (TH, T7451, Sigma, dil. 1:500) diluted in blocking solution were applied to the sections and incubated in a wet chamber overnight at 4 °C. After 3x washing in PBS, respective secondary antibodies were left in incubation for 1.5 h at room temperature: goat anti-mouse Cy2, dil. 1:250 (Jackson, West Grove, PA) and goat anti-rabbit Cy3, dil. 1:100 (Dianova, Hamburg, Germany). After 3x washing in PBS, cell nuclei were counterstained with DAPI for 2 min; after final washing in PBS, sections were dried at 37 °C for 10 min and embedded with Mowiol. From each brain region, namely cortex (CX), hippocampal CA3 region (CA3), hippocampal dentate gyrus (DG), deep mesencephalic reticular nucleus (DpMe), red nucleus (RN) and substantia nigra (SN), three sections per animal were stained and evaluated by a blinded investigator. In each region, the mean gray value was quantified by MATLAB scripts using the mean gray value of each specific region, normalized to the mean gray value of the negative control.

### Stereological Analysis of Dopaminergic and Non-dopaminergic Neurons in the SNpc

Immunohistochemical staining of dopaminergic and non-dopaminergic neurons in the SNpc and the stereological analysis of respective cell numbers were performed as described before (Saal et al. 2015; Tatenhorst et al. 2014; Tönges et al. 2012). Briefly, every fourth section of the substantia nigra was immunostained against TH and counterstained with Nissl. Then, the numbers of TH-positive and Nissl-positive/TH-negative neurons were evaluated by stereological counting by a blinded investigator, and results were compared between treatment groups.

### Statistical Analysis

The software used for the analysis was MATLAB 2013a Student version (The MathWorks Inc., Natick, MA) together with GraphPad Prism 6.01 (GraphPad Software Inc., La Jolla, CA). For the Kaplan–Meier curve of animal survival, SPSS statistic (version 24, IBM., Armonk, NY) was used. For group comparisons, ANOVA together with Tukey post hoc test was applied. The statistical test and the number of animals or experiments for each analysis are specified in the respective figure legends. Data are given as mean ± SEM. Differences were considered significant with *p* < 0.05 (*p < 0.05; **p < 0.01; ***p < 0.001).

## Results

### Fe-Induced Motor Impairment is Rescued by DFP Treatment

**Fig. 1:**
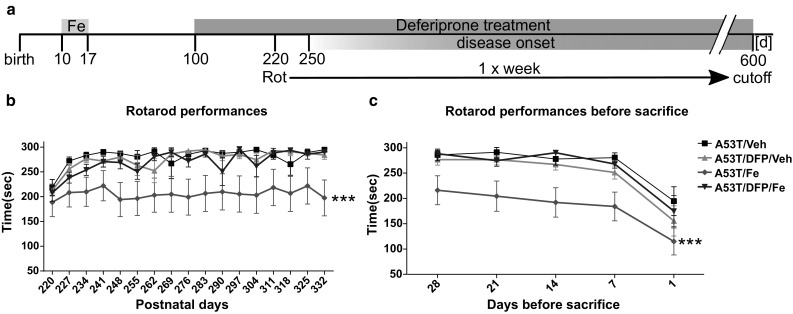
Rotarod analysis. **a** A53T mice were treated with 40 mg/kg bw Fe carbonyl for 8 consecutive days (from p10 to p17). At p100, animals were supplied with 10 mg/kg bw of the Fe chelator DFP. Rotarod was performed weekly starting from day 220. **b** Rotarod performances in the learning phase. The ANOVA with repeated measures and Tukey post hoc test showed a significant difference between A53T/Fe and the other groups (*p* < 1*10^−6^) **c**. Rotarod performance before mice were sacrificed. ANOVA with repeated measures and Tukey post hoc test (*p* < 1*10^−4^). The number of animals for each repetition is given in Supplementary Table 1

In order to assess the effects of Fe in the A53T mouse model on the behavioral level, animals were tested on the rotarod as well as on the Catwalk™ gait analysis system. The rotarod assessment was performed on transgenic mice once a week from p220 until they were sacrificed (Fig. 1a). The A53T/Veh animals learned to accomplish the task within the first 4 to 5 repetitions on the rotarod, and they stabilized their performances running in the average 283 ± 10 s on the device. The situation was similar to the animals of the A53T/DFP/Veh group that received DFP (271 ± 16 s). Conversely, the mice that had been treated with Fe in the early postnatal period displayed a learning curve that was not as steep as observed in the other groups, and they never reached a time of 300 s on the rotating rod (209 ± 7 s). Intriguingly, the effects of Fe supplementation were completely reversed by the DFP treatment: the average time on rotarod in the A53T/DFP/Fe group was 276 ± 10 s. One-way ANOVA for repeated measures followed by Tukey post hoc test revealed a highly significant difference (*p* < 1 × 10^−6^) between Fe-treated animals versus the other three groups (Fig. 1b). As the study progressed, the mice started to reach the disease onset stage and therefore the number of animal used for each repetition was progressively decreasing (see Supplementary Table 1). Similarly, the analysis of the last 5 weekly performances before animals were sacrificed revealed highly significant differences between the A53T/Fe and all other groups (*p* < 1 × 10^−4^; Fig. 1c). On the day of sacrifice , when all animals showed a maximal symptom load, the effects of the treatments were not distinguishable anymore (*p* = 0.29).

### DFP Improves Novel Object Recognition in Young Animals with Fe Intoxication

**Fig. 2:**
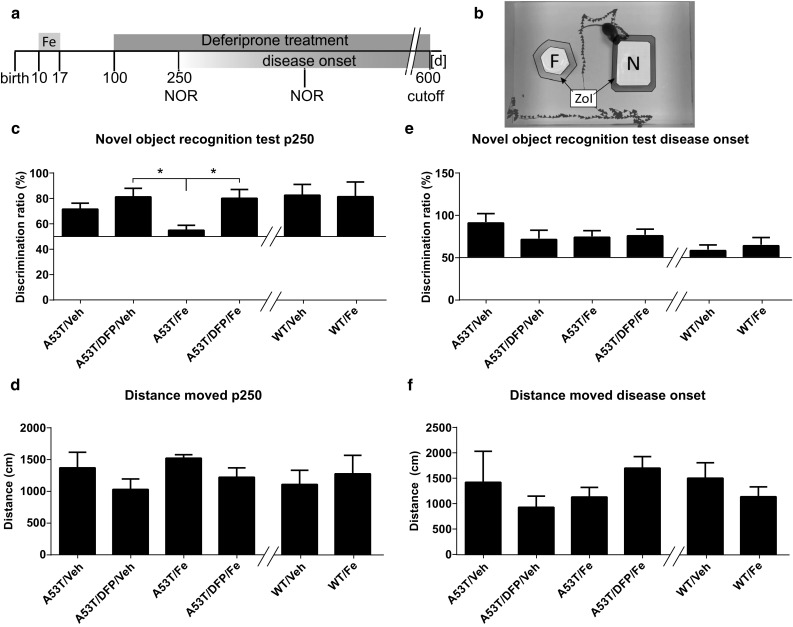
Novel object recognition test. **a**. A53T and WT littermate mice were treated with 40 mg/kg bw Fe carbonyl for 8 consecutive days (from p10 to p17), at p100 animals were supplied with 10 mg/kg bw of the Fe chelator deferiprone. NOR was performed at p250 and disease onset. Results are expressed as the ratio between the frequencies of nose directed to the novel object compared to both objects. **b**. Mice were put in an arena with a familiar (F) and a novel object (N). The time spent heading toward the objects when being in the zone of interaction (ZOI) was analyzed. **c**. NOR test performed at p250. The A53T/Fe animals perform significantly worse than the A53T/DFP/Veh animals (*p* = 0.042) and also worse than the A53T/DFP/Fe animals (value = 0.040) **d**. Distance run during the test at p250. **e**. NOR test performed at diseases onset. **f**. Distance run during the test at disease onset. One-way ANOVA with Tukey post hoc test. (*n* = 4 for A53T/Veh, *n* = 5 for the other groups at p250; *n* = 4 for A53T/Veh, *n* = 5 for A53T DPFctr, *n* = 8 for A53T/Fe and A53T/DFP/Fe at disease onset)

The NOR test is designed to evaluate especially novelty seeking behavior and memory in rodents. Healthy mice usually spend more time exploring a new object than a familiar one. The animals in this study were tested at p250 and at disease onset (Fig. 2a). At p250, A53T/Veh animals spent most of their time (71.56 ± 4.47%) exploring the new object in a similar fashion of A53T/DFP/Veh animals (81.24 ± 6.71%). However, A53T/Fe performed significantly worse (A53T/Fe 55 ± 3.88%) than A53T/DFP/Veh (one-way ANOVA over the transgenic mice performances with Tukey post hoc test, adjusted *p* = 0.042). Furthermore, also A53T/DFP/Fe animals spent significantly more time observing the novel object compared to the A53T/Fe animals (*p* = 0.040). There was no difference for the WT animals irrespective of the treatment received compared to each other. The distance moved was similar among the groups (Fig. 2d). When the mice were re-tested at disease onset, the differences observed at p250 leveled out (Fig. 2e). Still, at disease onset, the distance moved was not varied (Fig. 2f).

### Gait is Not Affected by Fe or DFP

**Fig. 3:**
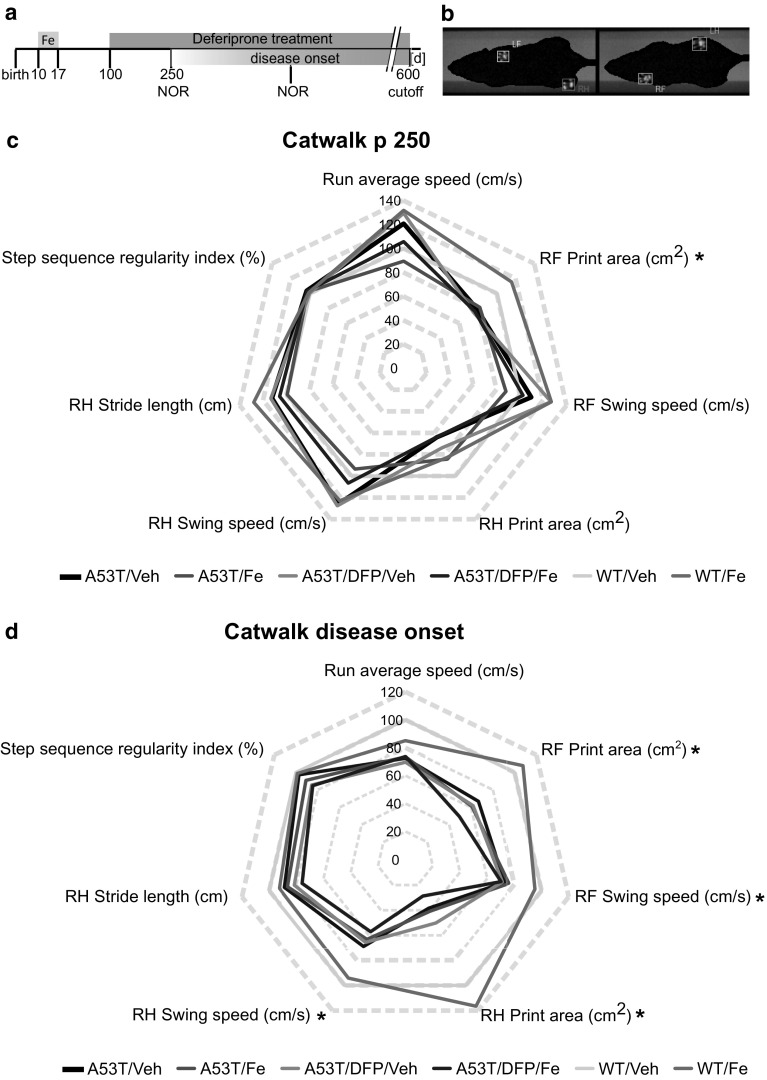
Catwalk™ gait analysis. **a**. A53T and WT littermate mice were treated with 40 mg/kg bw Fe carbonyl for 8 consecutive days (from p10 to p17), at p100 animals were supplied with 10 mg/kg bw of the Fe chelator DFP. Catwalk analysis was performed at p250 and disease onset. **b**. Example of footprint recording (RF = right front, LF = left front, RH = right hind, LH = left hind). **c**. Radar chart displaying the results of seven recorded gait parameters as difference in percent from the WT/Veh group at p250. *n* = 5 for all groups, except for A53T/DFP/Veh *n* = 4. **d**. Radar chart displaying the results of seven recorded gait parameters as difference in percent from the WT/Veh group at day of disease onset. *p < 0.05; A53T/Veh *n* = 4, A53T/DFP/Veh *n* = 5, A53T/Fe and A53T/DFP/Fe *n* = 8, WT/Veh *n* = 10

For the quantification of unforced motor behavior, Catwalk™ gait analysis system was used in order to evaluate the animals at p250 and at disease onset (Fig. 3a, b).

At p250, no statistically significant differences were observed in any of the parameters, except for the right front (RF) print area in which WT/Fe animals had a higher area compared to A53T mice (*p* = 0.049) (Fig. 3c). At the day of disease onset, most of the parameters chosen showed a significant difference between the A53T mice and the WT mice (RF print area *p* = 2 × 10^−6^; RF swing speed *p* = 0.047; right hind (RH) print area *p* = 2 × 10^−7^; RH swing speed *p* = 0.007). Treatment with Fe and/or DFP did not result in significant differences of the measured gait parameters (Fig. 3d).

### Overall Survival and Neuronal Cell Counts are Not Affected by Fe and DFP

**Fig. 4:**
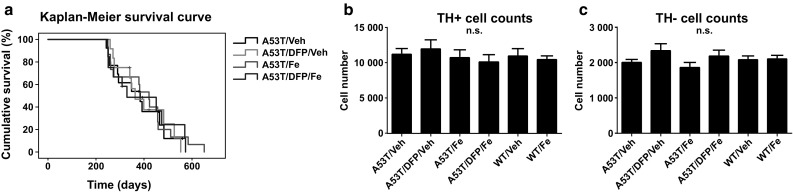
**a**. Kaplan–Meier survival curve for the A53T animals (*n* = 10 for all the groups; no significant differences). **b**. Quantification of TH-positive cells per substantia nigra (*n* = 5 for all the groups; no significant differences). **c**. Quantification of TH-negative/Nissl-positive neurons per substantia nigra (*n* = 5 for all the groups; no significant differences)

The Kaplan–Meier survival curve was calculated based on the disease onset time point (Fig. 4a).

The mean survival for each group was: A53T/Veh 379.43 ± 30.25 days; A53T/Fe 401.13 ± 32.45 days; A53T/DFP/Veh 398.23 ± 32.71 days; A53T/DFP/Fe 394.47 ± 40.10 days, no significant differences. Furthermore, also the stereological counting (Fig. 4b) demonstrated no statistically significant differences among the groups in the number of TH-positive neurons per SNpc (A53T/Veh 11,174 ± 641, A53T/DFP/Veh 11,928 ± 1044, A53T/Fe 10,700 ± 821, A53T/DFP/Fe 10,080 ± 296, WT/Veh 10,924 ± 875 and WT/Fe 10,418 ± 413). In addition to that, Nissl-positive/TH-negative neuron counts revealed no significant differences between the groups (A53T/Veh 1997 ± 50, A53T/DFP/Veh 2328 ± 150, A53T/Fe 1853 ± 97, A53T/DFP/Fe 2176 ± 138, WT/Veh 2072 ± 130 and WT/Fe 2093 ± 74) (Fig. 4c).

### Effects of Fe and DFP on aSyn Aggregation

**Fig. 5:**
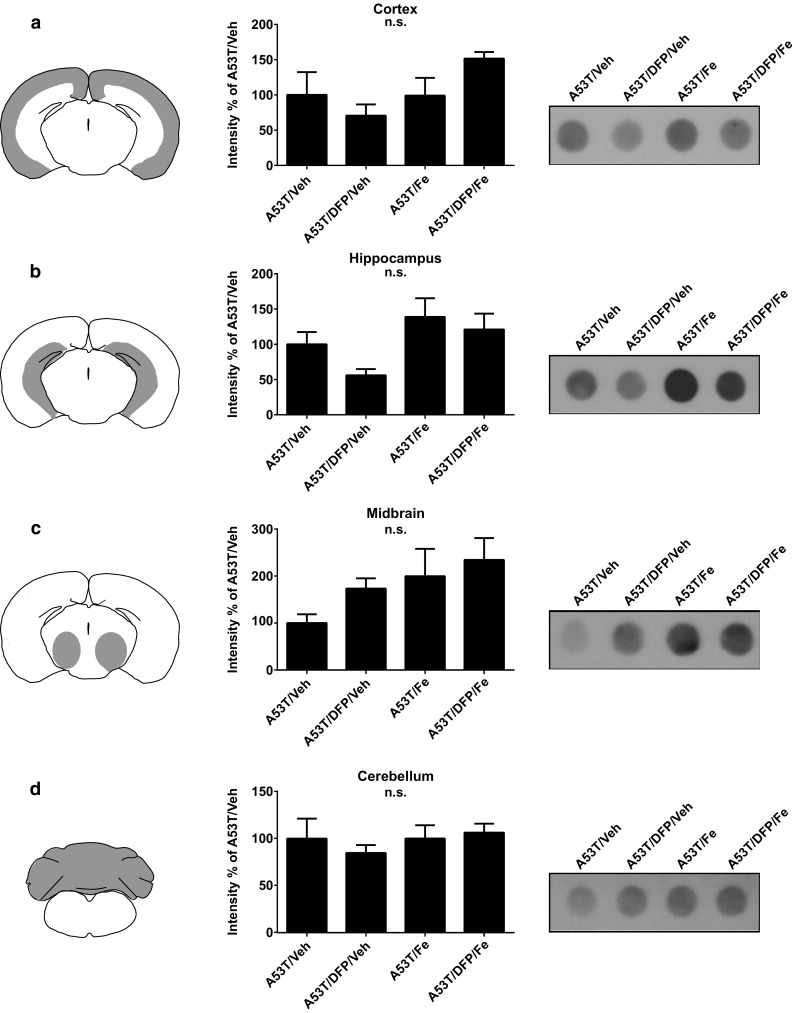
Dot blot assay for aggregated human aSyn in A53T mice. The tissue samples were homogenized and incubated for 10 min with proteinase K to digest the soluble forms of aSyn. Samples were blotted and revealed through an antihuman aSyn antibody to visualize proteinase K-resistant fraction. Each row shows a scheme of the analyzed area, the quantification of *n*=5 blots and an exemplary blot for **a**. cortex; **b**. hippocampus; **c**. midbrain; and **d**. cerebellum

In order to further characterize the interaction that occurs between Fe and aSyn in our model, we performed dot blot analysis after proteinase K-resistant digestion. We considered the proteinase K-resistant fraction as the aggregated forms of aSyn. Four brain regions were dissected for analysis: cortex, hippocampus, midbrain and cerebellum. Five animals per group of all the different A53T groups (A53T/Veh, A53T/DFP/Veh, A53T/Fe and A53T/DFP/Fe) were tested. After the digestion, only proteinase K-resistant aSyn was detected. WT animals did not show any proteinase K-resistant aSyn; therefore, they were therefore not included in the analysis (see Supplementary Fig. 1). Overall, no statistically significant differences between any of the analyzed groups were detected. In the cortex, however, there was a trend for less aggregation in the A53T/DFP/Veh group and more aggregation in the A53T/DFP/Fe group compared to the A53T/Veh group (*p* = 0.08) (Fig. 5a). In the hippocampus, again the A53T/DFP/Veh group showed a trend to less aggregation, while the A53T/Fe and A53T/DFP/Fe groups showed trends to more aggregation (*p* = 0.065) (Fig. 5b). No statistical differences or trends were observed in the midbrain and cerebellum areas (Fig. 5c, d).

**Fig. 6:**
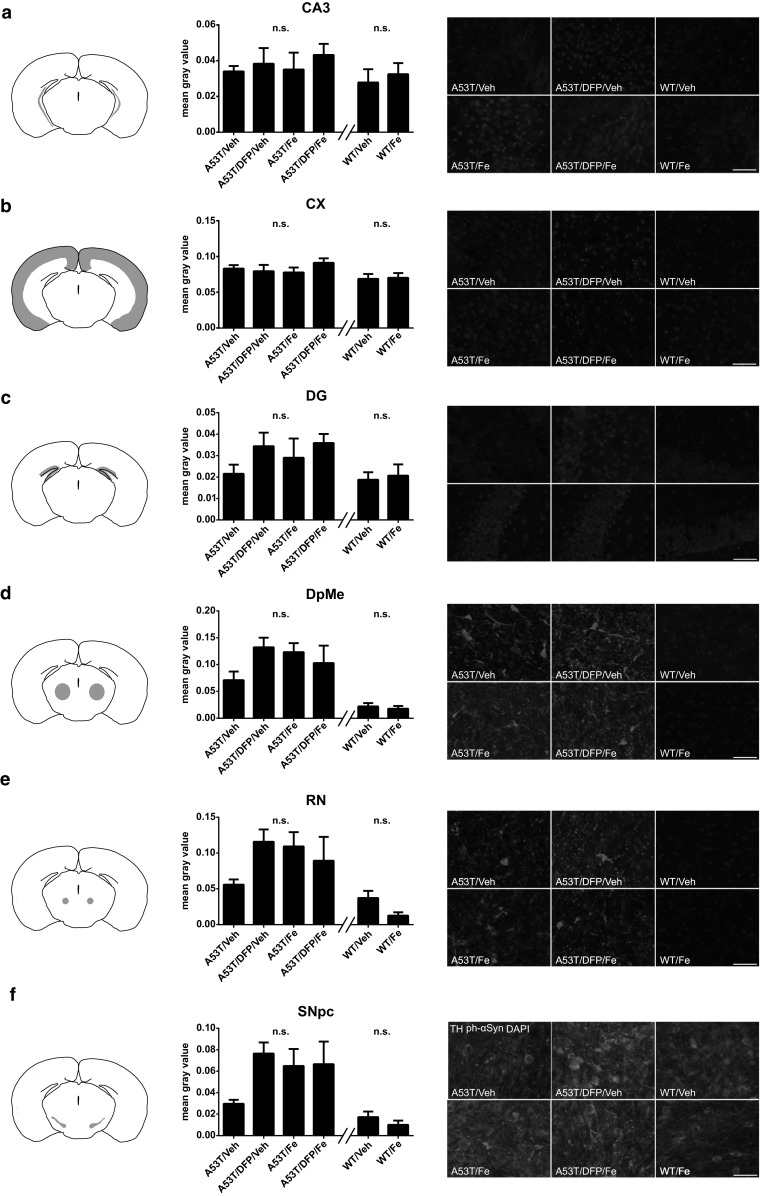
Histological evaluation of aSyn aggregation in A53T mice at disease onset using a p-S129-aSyn antibody for all the regions tested. This posttranslational modification was shown to be found in the aggregated form of aSyn only. Each row shows a scheme of the analyzed region, a quantification of p-S129-aSyn signal through fluorescence intensity (*n*=5 animals for each group), and representative photomicrographs. **a**. CA3 region; **b**. cortex (CX); **c**. dentate gyrus (DG); **d**. deep mesencephalic reticular nucleus (DpMe); **e**. red nucleus (RN); **f**. substantia nigra (SN). Scale bar = 20 µm

The histological analysis was performed to characterize in more detail the effects of Fe and DFP treatment on aSyn aggregation. The areas analyzed were: CA3, cortex (CX), dentate gyrus (DG); deep mesencephalic reticular nucleus (DpMe); substantia nigra (SN) and red nucleus (RN). The evaluation was done with antibodies against tyrosine-hydrolase to stain for dopaminergic neurons and antibodies against phosphorylated aSyn (p-S129-aSyn) as this form is found in aSyn aggregates. The results are expressed as mean gray values. Both, the hippocampus (CA3, DG) and the CX, displayed almost no reactivity for the p-S129-aSyn antibody, and the analysis of mean gray values confirmed no significant differences (Fig. 6a, b, c). The signals for p-S129-aSyn was much stronger in the analyzed midbrain regions (DpMe, RN, SN), but no significant differences were observed between the groups (Fig. 6d, e, f).

## Discussion

In the present study, we investigated the effects of moderate Fe exposure in an in vivo mouse model of aSyn aggregation. In detail, we quantified the effects of Fe supplementation on aSyn aggregation in vivo as well as its consequences on behavior. To counteract the effects of Fe overload, we employed the clinically licensed chelator DFP in a translational approach.

The survival of the animals involved in this study was similar to what has been already published for this model (Luk et al. 2012; Tatenhorst et al. 2016). This means that DFP and Fe administration, as well as their combination, was not detrimental for the lifespan of the animals. These findings were further corroborated by TH-positive and Nissl-positive/TH-negative neuron counts, which did not reveal any differences among the groups. Although the neuronal counts were not altered, we cannot exclude an involvement of striatal dopaminergic terminals. It is important to keep in mind that histology depicts the disease onset stage while we observed that the biggest behavior differences were predominantly in young mice. In contrast to our study, A53T mice had a decreased number of dopaminergic neurons at 8 months of age when comparing WT animals to A53T littermates after Fe supplementation (Billings et al. 2016). This difference is probably due to the higher Fe amounts administered to those animals.

We observed clear behavioral effects due to Fe presence in our paradigm. In fact, in the rotarod test, the animals of the control group (A53T/Veh) as well as the animals treated with DFP only (A53T/DFP/Veh) displayed a strong improvement in motor function within the first month, thus confirming that the DFP treatment was non-toxic for mice. In contrast, the performances of animals that have been treated with Fe suggested that the exposure to this metal reduced their learning abilities. Similar effects on the rotarod were reported in a murine model of neurodegeneration (Potter et al. 2011). Moreover, our findings are in agreement with a previous study, where a similar Fe load on wild-type mice produced impairments in performing the radial arm maze at 3 months of age (Fredriksson et al. 1999). Intriguingly, in our study DFP attenuated the detrimental effects of Fe exposure on mouse behavior. Actually, A53T/DFP/Fe mice performed the rotarod task as good as the control animals, which is in line with previous studies performed in rats (Sripetchwandee et al. 2014).

Furthermore, at p250 Fe-treated animals displayed a decreased novelty seeking behavior in NOR, thus pointing out a decline of their cognitive abilities. Importantly, this effect was again rescued by DFP treatment as the A53T/DFP/Fe animals were able to perform the NOR task as good as the A53T/DFP/Veh animals. At the time of disease onset, analysis of Catwalk™ data implied a severe gait impairment in transgenic A53T mice as compared to wild-type age-matched controls as described previously (Tatenhorst et al. 2016). The Catwalk system was, however, not able to detect any significant motor impairments induced by Fe or DFP treatment. This could be explained by the rather moderate Fe exposure paradigm used, which appears to induce effects on various cognitive domains in young animals (in the NOR test and in the learning curve for the rotarod), whereas motor function was not affected by the applied Fe dosage.

Next to behavioral characterization, we analyzed aSyn aggregation by dot blot (Angot et al. 2012). The analysis showed a trend for a reduction in aSyn aggregation for the animals treated with DFP (A53T/DFP/Veh group), both in the hippocampus and in the cortex. Furthermore, there was a trend for more aggregation in A53T animals supplemented with Fe, but the results did not reach statistical significance. Histological evaluation using a p-S129-aSyn antibody also failed to reveal significant differences among the different A53T mouse groups. The reason for this might be that both histological evaluations as well as dot blots were carried out at the stage of disease onset. Despite the variability shown due to inter-individual variations, the results were similar and therefore suggest that at disease onset the progression of the aggregation was also similar, independently of the possible modulation effects exerted by Fe or DFP. The dot blot analysis suggested a possible reduction in aSyn aggregation for animals that had been treated with DFP alone. This result goes in line with a previous publication, where a different chelator, clioquinol, was used in the A53T mouse model (Finkelstein et al. 2016). In that approach, the amount of SDS-insoluble aSyn after clioquinol treatment in brain lysates was significantly lower compared to controls. Another work carried out with the same transgenic mouse line, but with a higher Fe amount, showed that Fe at high concentrations could reduce the motoric functions of the A53T animals, which could not be rescued by clioquinol administration (Billings et al. 2016). Besides genetic models, clioquinol was also employed to reduce the oxidative stress caused by Fe in a MPTP mouse model of PD (Kaur et al. 2003). In this work, clioquinol administration increased the number of dopaminergic neurons and improved motor behavior. Remarkably, DFP has a very high affinity for Fe only (Galanello 2007), while clioquinol can chelate also other metals, such as Cu and Zn, with a greater affinity for the latter two as compared to Fe (Bareggi and Cornelli 2012; Colvin et al. 2008). This may affect not only aSyn aggregation and Fe chelation, but also several other cellular processes in which Cu and Zn are enzymatic cofactors. Unfortunately, the ability of clioquinol to bind Zn has been shown to induce subacute myelo-optic neuropathy in humans, complicating its translation to clinical use (Mao and Schimmer 2008).

It is well known that in PD pathology, free radicals and oxidative stress play a major role together with metal metabolism unbalance. MPTP-induced Parkinsonism in mice is essentially modeling oxidative stress and metabolic failure in PD. To this regard, an additional Fe supplementation to MTPT-treated mice could indeed exacerbate oxidative stress, leading to a depletion of dopaminergic neurons (Kaur et al. 2007). These findings support the idea that oxidative stress caused by unbound Fe can contribute greatly to the exacerbation of toxic effects of aSyn, regardless of its aggregation state (Funke et al. 2013). Therefore, the use of chelators has been proposed to hinder Fe toxicity (Weinreb et al. 2013). Oxidative stress is of importance also in familial forms of PD where genes other than aSyn are causal and where an increase in oxidative stress and mitochondrial impairment are known to contribute to the pathogenesis. As such, Parkin knockout mice that were also knock-in for the iron regulatory protein 2 displayed motor symptoms, showed mitochondrial damage and had a lower integrity of the neurons in the substantia nigra further underlining the connection of Fe metabolism in neurodegeneration (Asano et al. 2015). Also mutations in the leucine-rich repeat kinase 2 (LRRK2) in PD patients lead to an alteration of mRNA levels related to iron metabolism and cell survival pathways (Mutez et al. 2014). The Pink1 gene encodes for a serine/threonine protein kinase in the mitochondria, and it has also been linked to familial PD. In the SHSY-5Y cell line, it has been demonstrated that Pink1 contributes to oxidative stress through a Fe-dependent mechanism (Zhou et al. 2014). Taken together, the aforementioned data support the idea that normalizing Fe dyshomeostasis in the brain can be beneficial for PD patients, even when the etiopathology is not linked to aSyn aggregation itself. Iron chelators may thus also be of benefit in familial cases of PD.

Among chelators, DFP stands out as one of the most promising drugs for future therapies for PD. In fact, DFP is able to cross the blood–brain barrier (Fredenburg et al. 1996) and unlike other chelating drugs that deplete systemic Fe, DFP possesses conservative Fe chelation abilities. This means that DFP can redistribute the excess of intracellular Fe to the extracellular apotransferrin (Sohn et al. 2011), thus avoiding severe systemic Fe deprivation. A clinical phase-I trial conducted in a small cohort of PD patients further suggested that DFP has indeed a beneficial effect on patients’ motor symptoms measured by the Unified Parkinson’s Disease Rating Scale (UPDRS), when administrated as early as possible after diagnosis (Devos et al. 2014). Currently, two phase-II trials (NCT02655315 and NCT02728843) are ongoing with a higher number of patients involved. Taken together, all these studies together with ours endorse the use of DFP in the near future for PD treatment.

In summary, we provided an in vivo mouse model, in which we aimed to visualize effects of moderate Fe supplementation on aSyn aggregation. This model reproduced peculiar PD characteristics such as aSyn aggregation and gait impairments. Our study proved that the synergistic effects of Fe and aSyn aggregation cause behavioral impairments, especially with regard to memory and learning. The Fe-induced impairments were successfully rescued by DFP, thus supporting the clinical potential of Fe chelation in PD.

## Electronic supplementary material

Below is the link to the electronic supplementary material.

Supplementary Figure 1 Supplementary material 1 (EPS 1306 kb) Dot-blot analysis of transgenic A53T mice sacrificed at the day of disease onset (a) and of age-matched wildtype littermates (b). Detection was performed with anti-aSyn antibody with the same exposure time. The top line dots show the undigested homogenates while the bottom part shows the homogenates after 10 min of incubation with proteinase K. Western blot analysis of homogenates incubated with increasing times with proteinase K (c). Soluble proteins were digested by proteinase-K. No GAPDH could be detected after incubation with the enzyme

Supplementary Figure 2 Weight of the animals during rotarod stratified by sex (M=male; F=female). (a.) Average weight of mice tested on the rotarod between postnatal day 220 and 332. Two-way ANOVA showed no significant differences among the groups. (b.) Average weight of mice tested for rotarod before sacrifice due to disease onset. Two-way ANOVA displayed significant differences among the groups over time showing weight decrease due to the disease onset (*p* = 0.0043), but also between the groups (*p* = 0.012) as the groups treated with Fe (independently from the sex) were heavier than the others before disease onset. Please note that despite the weight, the animals in the group A53T/DFP/Fe are able to perform as good as the other groups and there is no weight difference between the A53T/Fe and A53T/DFP/Fe groups (EPS 168 kb)

Supplementary Table 1 Number of animals used in the rotarod evaluation at each time point. In the course of the experiment the number of animals decreases due to the onset of the disease (EPS 74 kb)
